# The Influence of Dimerization on the Pharmacokinetics and Activity of an Antibacterial Enzyme Lysostaphin

**DOI:** 10.3390/molecules24101879

**Published:** 2019-05-16

**Authors:** Alexander V. Grishin, Natalia V. Lavrova, Alexander M. Lyashchuk, Natalia V. Strukova, Maria S. Generalova, Anna V. Ryazanova, Nikita V. Shestak, Irina S. Boksha, Nikita B. Polyakov, Zoya M. Galushkina, Lyubov A. Soboleva, Sergey S. Vetchinin, Vitaliy M. Pavlov, Anna S. Karyagina, Vladimir G. Lunin

**Affiliations:** 1 N. F. Gamaleya National Research Center of Epidemiology and Microbiology, Ministry of Health of the Russian Federation, 123098 Moscow, Russia; nvlavr@mail.ru (N.V.L.); lamy13@mail.ru (A.M.L.); natalka.fanr@gmail.com (N.V.S.); keysi1986@mail.ru (M.S.G.); laurikim91@mail.ru (A.V.R.); boksha_irina@mail.ru (I.S.B.); polyakovnb@gmail.com (N.B.P.); gazoya@yandex.ru (Z.M.G.); l.a.soboleva@yandex.ru (L.A.S.); akaryagina@gmail.com (A.S.K.); lunin1955@gmail.com (V.G.L.); 2 All-Russia Research Institute of Agricultural Biotechnology, Russian Academy of Sciences, 127550 Moscow, Russia; 3 M.V. Lomonosov Institute of Fine Chemical Technologies, MIREA Russian Technological University, 119048 Moscow, Russia; nikita1305@mail.ru; 4 Mental Health Research Center, 115522 Moscow, Russia; 5 Vernadsky Institute of Geochemistry and Analytical Chemistry, Russian Academy of Sciences, 119991 Moscow, Russia; 6 State Research Center for Applied Microbiology and Biotechnology, 142279 Obolensk, Russia; vetchinin@obolensk.org (S.S.V.); vitpav@obolensk.org (V.M.P.); 7 A. N. Belozersky Institute of Physico-Chemical Biology, M.V. Lomonosov Moscow State University, 119992 Moscow, Russia

**Keywords:** antibiotic resistance, endolysin, lysin, lysostaphin, dimerization, pharmacokinetics, staphylococcus

## Abstract

The increasing prevalence of antibiotic-resistant strains of pathogenic bacteria is a major healthcare problem. Antibacterial lysins are enzymes that cleave the peptidoglycan of the bacterial cell wall. These proteins hold potential as a supplement or an alternative to traditional antibiotics since they are active against antibiotic resistant strains. However, antibacterial lysins are rapidly eliminated from the systemic circulation, which limits their application. Dimerization of an anti-pneumococcal lysin Cpl-1 has been demonstrated to decrease the clearance rate of this protein in mice. In the present work, we constructed a dimer of an anti-staphylococcal lysin lysostaphin by fusing it with an anti-parallel α-helical dimerization domain. Lysostaphin dimer had a more favorable pharmacokinetic profile with increased terminal half-life and area under the curve (AUC) values compared to monomeric lysostaphin. However, the staphylolytic activity of dimerized lysostaphin was decreased. This decrease in activity was likely caused by the dimerization; since the catalytic efficacy of lysostaphin dimer towards pentaglycine peptide was unaltered. Our results demonstrate that, although dimerization is indeed beneficial for the pharmacokinetics of antibacterial lysins, this approach might not be suitable for all lysins, as it can negatively affect the lysin activity.

## 1. Introduction

The emergence and spread of antibiotic-resistant bacterial strains is a growing concern for modern healthcare [1,2]. The most prominent pathogens with a high prevalence of antibiotic-resistant strains have been included into the so-called ESKAPE group [3] that consists of *Enterococcus* species resistant to vancomycin [4], methicillin-resistant *Staphylococcus aureus* [5], *Klebsiella pneumoniae*, *Acinetobacter baumanii*, *Pseudomonas aeruginosa*, and *Enterobacter* spp. overexpressing β-lactamases and efflux pumps [6,7]. While novel antibiotics are urgently needed to combat drug-resistant bacteria, creating an ideal antibiotic is a challenging task [1,2,8]. Many different approaches have been followed so far, including β-lactamase inhibitors, efflux pumps inhibitors [9,10,11], antivirulence compounds that block virulence factors instead of killing bacteria [12], novel modifications of traditional antibiotics, antimicrobial peptides, quorum sensing inhibitors, and others [11]. Among other options, antibacterial lysins have gained increased attention. These enzymes cleave peptidoglycan in the bacterial cell wall, which leads to osmotic lysis and death of bacterial cells. Antibacterial lysins possess a number of important advantages over traditional antibiotics, such as high potency, rapid action, the lower propensity for resistance development and the ability to eradicate biofilms [13,14]. Importantly, most lysins consist of one or more catalytic domains, and one or more peptidoglycan-binding domains. Combining catalytic and peptidoglycan-binding domains from different lysins can help create chimeric lysins with specificity towards different bacterial species [15]. Recently, the safety of two anti-staphylococcal lysins, SAL-1 and CF-301, upon systemic administration was studied in phase I clinical trials. Both lysins were given by intravenous infusions in doses ranging from 0.1 to 10 mg/kg (SAL-1) and 0.04 to 0.4 mg/kg (CF-301), and only mild and transient adverse effects were reported [16,17].

A significant drawback of antibacterial lysins is their rapid elimination from systemic circulation. This fast elimination is characteristic of relatively small therapeutic proteins, and is caused by the active excretion through the kidneys [18]. The estimated serum half-life of anti-staphylococcal lysin lysostaphin in mice was less than 1 h [19], and the half-life times of anti-pneumococcal lysins Cpl-1 and LytA were measured to be 20.5 and 22.5 min, respectively [20,21]. In humans, a similar elimination rate was observed for SAL-1 (half-life between 0.04 and 0.38 h), although lysins P128 and CF-301 were cleared from the circulation appreciably slower, with half-lives of ~5 h and 11.3 h, respectively [22]. The fast elimination rate of antibacterial lysins leads to the need of multiple administrations and/or larger doses of the protein to achieve bacterial eradication [23,24]. Thus, it would be desirable to create lysin variants with increased residence time in the systemic circulation.

The most common technique of improving the pharmacokinetic parameters of therapeutic proteins is the conjugation with polyethylene glycol (PEGylation) [25]. Recently, however, concerns about the safety of PEGylated pharmaceuticals have been raised. Anti-PEG antibodies and hypersensitivity reactions to PEG have been reported, along with cell vacuolation and PEG accumulation in different tissues [26]. More importantly, the bulky PEG chain often negatively influences the specific activity of the protein of interest. This negative effect of PEGylation was demonstrated for antibacterial lysins as well [19,27]. Conjugation of lysostaphin with 40 kDa branched PEG resulted in the increase of serum half-life from less than 1 h for unmodified lysostaphin to 24 h. However, conjugation of more than one PEG chain greatly reduced the lysostaphin antibacterial activity [19]. Similarly, PEGylation of anti-pneumococcal lysin Cpl-1 by a single PEG chain completely abolished the bacteriolytic activity of the enzyme, regardless of the PEG chain size [27].

PEGylation decreases the elimination rate by increasing the hydrodynamic volume of the protein and limiting the efficacy of kidney filtration. An alternative approach to increasing the hydrodynamic volume of a protein is multimerization. This strategy was successfully applied to various proteins, including many antibodies, erythropoietin, thrombopoietin-mimetic peptide, extracellular domains of IL-1 and VEGF receptors, recombinant factors IX and VIII [28,29,30], as well as antibacterial lysins. In the work of Resch et al. [31], a cysteine residue was introduced into the C-terminal region of Cpl-1 lysin to allow the disulfide bond formation between two protein molecules. This modification did not influence the activity of the lysin, but led to a ten-fold decrease in the clearance rate from murine plasma. Interestingly, when the cysteine residue was placed in positions other than the C-terminus, dimerization abolished the Cpl-1 antibacterial activity. The authors speculate that Cpl-1 naturally forms homodimers upon interaction with the bacterial cell, and this dimerization is mediated by a 10 amino acid stretch at the lysin C-terminus. If this is the case, the disulfide-linked Cpl-1 dimer mimics the natural form of this protein.

In this work, we created a dimerized version of lysostaphin, another antibacterial lysin, by fusing genes coding for lysostaphin and an antiparallel α-helical dimerization domain, and investigated the influence of dimerization on its pharmacokinetic properties and bacteriolytic activity. Dimerization indeed prolonged the residence time of lysostaphin in the systemic circulation, but considerably diminished its bacteriolytic activity. We thus conclude that dimerization cannot be considered a universal strategy for improving the pharmacokinetic characteristics of antibacterial lysins.

## 2. Results and Discussion

### 2.1. Creation of a Dimerized Version of Lysostaphin

**Figure 1:**
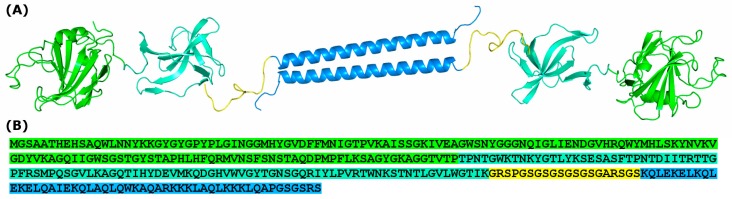
3D model and amino acid sequence of Lst-HDD. (**A**) A theoretical model of Lst-HDD homodimer. Lysostaphin catalytic domain is colored green, lysostaphin peptidoglycan-binding domain is colored cyan, dimerization domain is colored blue and spacer is colored yellow. The model was constructed in PyMOL (Schrödinger LLC, USA) based on PDB entry 4LXC. (**B**) The amino acid sequence of Lst-HDD. The color scheme as in (**A**).

We have chosen lysostaphin as one of the most active and well-studied antibacterial enzymes. Lysostaphin is a protease produced by *Staphylococcus simulans* that cleaves pentaglycine cross-bridges in the *Staphylococcus aureus* peptidoglycan [32]. Similarly to many other antibacterial lysins, lysostaphin contains an N-terminal catalytic domain and a C-terminal peptidoglycan binding domain. Unlike Cpl-1, lysostaphin was not shown to dimerize or require dimerization for activity. Thus, it is interesting to measure the effect of dimerization on lysostaphin activity and pharmacokinetics. We constructed a recombinant fusion protein consisting of lysostaphin and a homodimerization domain connected by a glycine-serine spacer (Figure 1). This protein is further referred to as Lst-HDD. The dimerization domain used in this study is a 44 amino acids long α-helix designed to form dimers in an antiparallel orientation [33].

**Figure 2:**
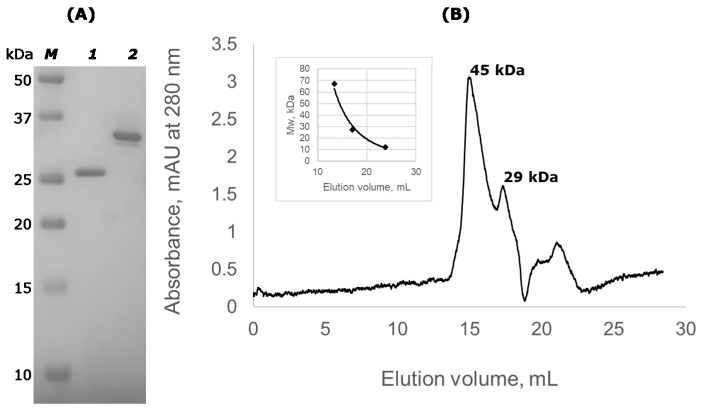
SDS-PAGE and size exclusion chromatography (SEC) of Lst-HDD. (**A**) SDS-PAGE of purified lysostaphin (lane 1) and Lst-HDD (lane 2); lane M contains molecular weight standards with their molecular weights indicated on the left (**B**) SEC chromatogram of Lst-HDD. The inlet shows the molecular weight calibration of the column.

Recombinant lysostaphin and Lst-HDD were produced in *Escherichia coli* and purified by ion-exchange chromatography to apparent homogeneity (Figure 2A). The sequence of purified Lst-HDD corresponded to the theoretical sequence, which was confirmed by mass spectrometry analysis of protein fragments after trypsinolysis.

### 2.2. Lst-HDD Predominantly Exists as A Dimer

To test the ability of Lst-HDD to form dimers in the solution, we performed size exclusion chromatography (SEC) (Figure 2). SEC demonstrated that 75 ± 2.2% of the protein eluted in the volume corresponding to the relative molecular mass of 45 kDa, 18 ± 2.5% eluted in the 29 kDa region and a small portion of impurities eluted as ~16 kDa. The first two peaks likely correspond to the dimeric and monomeric species of Lst-HDD, respectively. Although their calculated molecular masses are ~70 and ~35 kDa, the difference can probably be attributed to the supposedly elongated shape of Lst-HDD causing it to elute faster than globular proteins of respective molecular weight. Thus, Lst-HDD was indeed able to dimerize, although not all molecules formed dimers. However, the mobile phase in the size exclusion chromatography experiments contained 0.5 M NaCl. The high concentration of salt weakens electrostatic interactions. Since electrostatic interactions play a significant role in the dimerization of the selected dimerization domain [33], a higher proportion of dimerized Lst-HDD molecules are expected under low-salt conditions. Alternatively, the reason for incomplete dimerization of Lst-HDD could be the improper folding of a part of the molecules.

### 2.3. Bacteriolytic But Not the Catalytic Activity of Lysostaphin Is Affected by Dimerization

Unlike lysostaphin that was synthesized in soluble form, the Lst-HDD protein was purified from inclusion bodies. To see if the enzymatic activity of the protein was affected by the purification procedure and/or by the addition of the dimerization domain, we tested the ability of both Lst-HDD and lysostaphin to hydrolyze pentaglycine peptide. The enzyme solutions were mixed with pentaglycine and incubated at 37 °C. The extent of pentaglycine hydrolysis was estimated spectrophotometrically after the reaction with ninhydrin. Ninhydrin reacts with amines with the formation of a colored product, and the amount of free amino groups in the solution doubles upon the hydrolysis of pentaglycine into di- and triglycine, increasing the color intensity after ninhydrin treatment. Both proteins hydrolyzed pentaglycine with identical efficacy. The optical density increased at the rate of 0.013 ± 0.004 h^−1^ with 1 µM Lst and 0.013 ± 0.002 h^−1^ with 1 µM of Lst-HDD. Thus, the purification of Lst-HDD from inclusion bodies yielded protein with intact catalytic activity.

Next, we compared the antibacterial activity of Lst-HDD and lysostaphin. Minimum inhibitory concentration (MIC) of Lst-HDD towards *S. aureus* ATCC 29,213 was 3.2 µg/mL. While this value is in the range of MICs of several antibiotics [34], it is 32 times higher than the MIC of lysostaphin against the same strain in our experiments—0.1 µg/mL. Thus, dimerization reduced the antibacterial activity of lysostaphin to a considerable extent.

**Figure 3:**
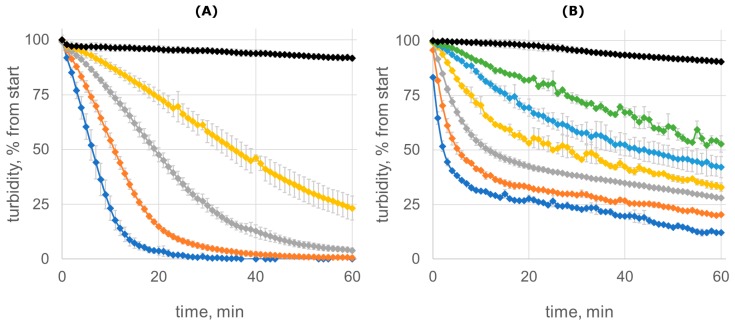
Clearing of *S. aureus* ATCC 29,213 cell suspension due to different concentrations of lysostaphin (**A**) or Lst-HDD (**B**). Lysostaphin concentrations are 2 µg/mL (blue), 1 µg/mL (orange), 0.5 µg/mL (grey), 0.25 µg/mL (yellow), and control without lysostaphin (black); Lst-HDD concentrations are 12 µg/mL (blue), 6 µg/mL (orange), 3 µg/mL (grey), 1.5 µg/mL (yellow), 0.75 µg/mL (light blue), 0.38 µg/mL (green), and control without Lst-HDD (black). The mean values of *n* = 3 (lysostaphin) or *n* = 2 (Lst-HDD) experiments are shown, error bars represent standard deviation.

The staphylolytic activities of Lst-HDD and lysostaphin were further compared by monitoring the reduction of the turbidity of staphylococcal cell suspension (Figure 3). We measured the time required to reduce the turbidity of the cell suspension by half by different concentrations of the enzymes and then adjusted it to 50 nM for each protein to allow direct comparison. In this experiment, Lst-HDD demonstrated decreased activity compared to lysostaphin, although the difference was not as dramatic as in the case of MIC. Lysostaphin decreased the turbidity of the suspension by half in 8.4 ± 0.9 min, while Lst-HDD needed 19.8 ± 1.5 min to achieve the same result. More importantly, the shape of Lst-HDD turbidity curves was very different from that of lysostaphin and was characterized by a fast initial decrease in the turbidity that slowed down noticeably as the reaction progressed (Figure 3B). The effect was most prominent at higher enzyme concentrations.

Thus, dimerization of lysostaphin had no effect on its catalytic efficacy but decreased its staphylolytic activity, which was evident from both MIC and turbidity measurements. Apparently, dimerization negatively affected the interaction of Lst-HDD with *S. aureus* cell wall peptidoglycan. This could probably be explained by the slower penetration of Lst-HDD into deep peptidoglycan layers, which would limit the action of the enzyme to the outer region of the cell wall and decrease the cell lysis efficacy. On the other hand, the presence of the dimerization domain could reduce the ability of lysostaphin to bind the peptidoglycan due to the introduced steric hindrance, as it was observed for PEGylated lysostaphin [19]. However, neither of these hypotheses explains the fast lysis of *S. aureus* cells by Lst-HDD at the early stages of the reaction (Figure 3B). It is conceivable that lysostaphin binds the peptidoglycan very tightly and only slowly disengages from the cell wall debris after the cell lysis. This would deplete the enzyme as the reaction progresses and slow down the lysis of the remaining cells. Some *Listeria* lysins were shown to bind the cell wall with high affinity and proposed to be such “one-use” enzymes [13]. Since Lst-HDD dimer has two peptidoglycan-binding domains, it should bind the peptidoglycan tighter, which might lead to enhanced binding rate in the beginning, faster enzyme depletion and slower lysis at the later stages of the reaction. Recently, the lysostaphin peptidoglycan-binding domain was shown to bind staphylococcal cells with a moderate affinity of 1–2 µM [35]. However, this value was measured for the peptidoglycan-binding domain with GFP fused to its C-terminus, which might affect its interaction with the peptidoglycan. Interestingly, when Cpl-1 lysin was dimerized tail-to-tail by a C-terminal disulfide bridge, its antibacterial activity was unaffected [31]. However, Cpl-1 is thought to naturally dimerize upon binding to the peptidoglycan [31]. On the contrary, lysostaphin normally acts in monomeric form. This difference might explain the decrease in lysostaphin activity upon dimerization.

### 2.4. Dimerization Improves the Pharmacokinetic Characteristics of Lysostaphin

**Figure 4:**
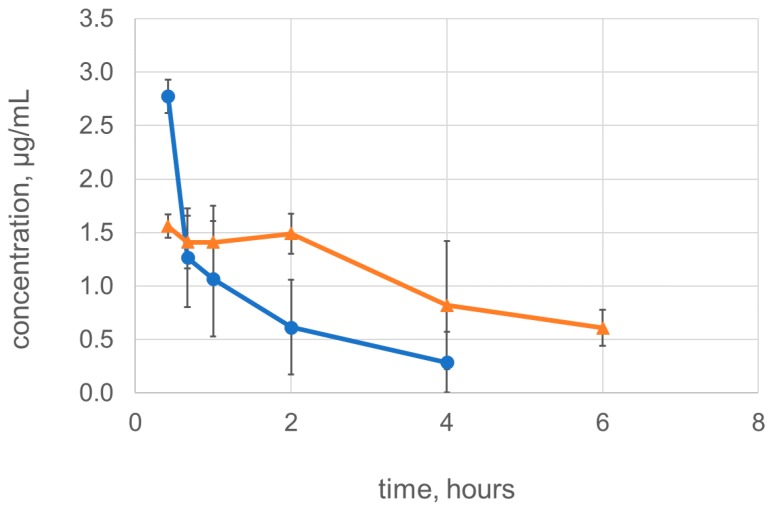
Residual concentrations of lysostaphin (blue, circles) and Lst-HDD (orange, triangles) in rat plasma. The mean values over *n* = 4 rats are shown, error bars represent standard deviation.

Finally, we tested if dimerization of lysostaphin increased its residence time in the systemic circulation. The rats received bolus intravenous injections of 500 µg of lysostaphin or Lst-HDD and the residual concentrations of the enzymes in plasma were measured using ELISA (Figure 4). Lysostaphin was quickly eliminated from rat plasma with a terminal half-life of 1.5 ± 0.7 h, similarly to what was previously described [19]. The terminal half-life of Lst-HDD increased compared to lysostaphin and amounted to 3.1 ± 0.6 h (*p* = 0.01). Similarly, the area under the curve (AUC) of Lst-HDD was greater than AUC of lysostaphin (9.1 ± 2.8 mg h/L vs. 3.5 ± 2.4 mg*h/L, respectively, *p* = 0.02). This difference was smaller than the tenfold difference in the AUC achieved by the dimerization of Cpl-1 [31]. The theoretically calculated concentration of the proteins in plasma after the injection was ~50 µg/mL. However, there was only 2.8 ± 0.2 µg/mL of lysostaphin and 1.6 ± 0.1 µg/mL of Lst-HDD left at the first sampling time point 25 min post-injection. Thus, ~95% of both lysostaphin and Lst-HDD appeared to have been eliminated by the time of the first blood sampling. Apparently, the elimination of lysostaphin and Lst-HDD follows a biphasic pattern, with quick elimination of the protein right after the injection that is slowed down after 30–45 min. A similar pattern was observed earlier for lysostaphin and Cpl-1 lysin [19,31]. Interestingly, although both the covalent dimerization of Cpl-1 and non-covalent dimerization of lysostaphin exerted a beneficial effect on the pharmacokinetic characteristics of the proteins, this effect appears to be caused by different factors. Covalent dimerization of Cpl-1 slowed down the elimination right after the injection, but did not seem to affect the terminal half-life (see Figure 4 in [31]). On the contrary, non-covalently dimerized Lst-HDD was cleared at the same rate or even faster than monomeric lysostaphin in the beginning but had the increased half-life in the terminal elimination phase. The cause for this discrepancy is unclear. It can be caused by the covalent vs. non-covalent nature of Cpl-1 and lysostaphin dimers. However, a more probable explanation is the difference in the net charge between Cpl-1 and lysostaphin. Lysostaphin and Lst-HDD are highly positively charged molecules; Lst-HDD has a net positive charge of +21. On the contrary, Cpl-1 carries a net negative charge of –16. The glomerular filter is negatively charged and known to excrete positively charged molecules more efficiently [36]. When Cpl-1 is dimerized, the increase in molecular weight and the net negative charge both lead to the less efficient glomerular filtration. However, when lysostaphin is dimerized, the increase in molecular weight that prevents glomerular filtration is accompanied by the increase in the net positive charge, which stimulates the filtration. The interplay between these two opposing effects might result in the observed pharmacokinetic curve of Lst-HDD. On the other hand, one cannot exclude the possibility that Lst-HDD non-specifically interacts with coiled-coil proteins in blood or tissues through its α-helical dimerization domain, which might further complicate the elimination profile of Lst-HDD.

## 3. Materials and Methods

### 3.1. Cloning, Expression, and Purification

The pQE6-based plasmid pL330 coding for recombinant lysostaphin was obtained previously [37]. DNA fragment coding for the full sequence of Lst-HDD flanked by NcoI and Kpn2I restriction sites was synthesized by Evrogen (Evrogen JSC, Moscow, Russia) and cloned into pQE6 plasmid resulting in plasmid pL473. The plasmids were transformed into *E. coli* M15 for protein production.

Lysostaphin was produced and purified essentially as described before [23]. Briefly, the *E. coli* strain M15 carrying pL330 plasmid was grown in 500 mL flasks containing 200 mL LB medium (5 g/L yeast extract, 10 g/L tryptone, and 10 g/L NaCl) supplemented with 25 mg/L kanamycin and 150 mg/L ampicillin at 37 °C and 180 rpm. When the optical density (OD) reached 1.0, the recombinant protein expression was induced by the addition of IPTG to the final concentration of 0.5 mM, and the culture was further incubated for 3 h. The cells were harvested by centrifugation at 11,285× *g* and 4 °C for 10 min. The cells were resuspended in lysis buffer (10 mM Tris-HCl, pH 7.5, with 50 mM NaCl and 50 µg/mL lysozyme [AppliChem Panreac, Darmstadt/Barcelona, Germany/Spain]) and sonicated for 2 min on ice (5 s pulses with 3 s intervals, amplitude 60%; Bandelin Sonopuls HD3200, Bandelin Electronic, Berlin, Germany). The suspension was centrifuged for 35 min at 10,000× *g* and 4 °C, and the supernatant was used for protein purification. The supernatant was loaded onto a WorkBeads 40S cation exchange column (Bio-Works Technologies, Uppsala, Sweden) equilibrated with 10 mM Tris-HCl buffer, pH 7.5, with 50 mM NaCl. The column was washed with 20 column volumes of the same buffer and lysostaphin was eluted with a NaCl gradient from 50 to 500 mM in 10 mM Tris-HCl, pH 7.5 in 5 column volumes.

Lst-HDD was produced following the same procedure as for lysostaphin. However, since Lst-HDD was almost exclusively found in inclusion bodies, the purification procedure had to be modified. The pellet obtained after the lysed biomass centrifugation was washed twice with the water solution of 1 M urea, 1% Triton X100, and 500 mM NaCl. The pellet was dissolved in the 10 mM Tris-HCl buffer, pH 7.5, containing 8 M urea and 50 mM NaCl, clarified by centrifugation at 16,400× *g* and 4 °C for 20 min and loaded onto WorkBeads 40S column equilibrated with the same buffer. The column was washed with 20 column volumes of the same buffer and 10 column volumes of the buffer containing 10 mM Tris-HCl and 50 mM NaCl, pH 7.5, to remove urea. Lst-HDD was eluted with a NaCl gradient from 50 to 700 mM in 10 mM Tris-HCl, pH 7.5 in 5 column volumes. Eluted protein fractions were pooled, precipitated by 50% saturated ammonium sulfate, and centrifuged. The pellet was dissolved in distilled water, dialyzed against distilled water to remove ammonium sulfate and centrifuged at 16,400× *g* and 4 °C for 20 min to remove undissolved protein aggregates. The obtained supernatants were aliquoted and stored at −70 °C.

To assess the purity of lysostaphin and Lst-HDD they were subjected to Laemmli SDS-PAGE in 15% polyacrylamide gel. Before electrophoresis, the protein samples were mixed with the reducing Laemmli sample buffer with DTT and heated at 95° for 5 min. The Precision Plus Protein™ Dual Color Standards (Bio-Rad Laboratories Inc, Hercules, CA, USA) molecular weight standards were used. The gel was stained with Coomassie Brilliant Blue and imaged using GelDoc XR+ gel documentation system (Bio-Rad Laboratories Inc, Hercules, CA, USA).

The protein concentrations were determined using BCA microplate assay (Applichem Panreac, Darmstadt/Barcelona, Germany/Spain) with bovine serum albumin (BSA) (Sigma-Aldrich, St. Louis, MO, USA) as a standard.

Mass-spectrometry analysis of Lst-HDD was performed on the protein band excised from SDS-PAGE gel. In-gel digestion was performed according to Shevchenko et al. [38] with minor modifications. Briefly, the excised protein band was reduced with DTT, alkylated by iodoacetamide and treated with trypsin. To extract the peptides from the gel the specimen was first sonicated for 15 min in extraction buffer 1 (50% acetonitrile, 5% formic acid in MilliQ water), then in extraction buffer 2 (90% acetonitrile, 5% formic acid in MilliQ water) using an ultrasonic bath (Bandeline Sonorex, Bandeline electronics, Berlin, Germany). The supernatants were collected, merged, dried under vacuum, and reconstituted in 0.1% formic acid.

The solution of tryptic peptides (2 µL) in 0.1% formic acid was mixed with 0.25 µL of 2.5 dihydrobenzoic acid (25 mg/mL in 30% solution of acetonitrile containing 0.5% acetic acid, *v*/*v*) on a steel target. The mixture was air dried at 23 °C. The peptides were analyzed using a time-of-flight UltrafleXtreme mass spectrometer (Bruker Daltonics, Bremen, Germany) with a MALDI source equipped with a Smartbeam laser (Nd:YAG, wavelength 355 nm, frequency 2 kHz) in a regime of detection of positive ions using a reflectron at the following settings of ionic source: 20.12 kV on the ion source IS1, 17.82 kV—on IS2, 7.46 kV—on lenses, 21.05 kV—on reflectron Ref1, and 10.80 kV—on Ref2. Ions were detected in the mass range from 700 to 5000 Th. Peaks corresponding to autolytic fragments of trypsin and keratin were used as internal standards and excluded from the list of detected masses.

### 3.2. Size Exclusion Chromatography

Size exclusion chromatography was performed at room temperature on AKTA FPLC using ENrich™ 650 High-Resolution Size Exclusion Column (Bio-Rad Laboratories Inc, Hercules, CA, USA) in 50 mM Tris-HCl, 500 mM NaCl, pH 7.5 as mobile phase, at 0.35 mL/min flow rate. Lst-HDD (100 µL) was injected in a concentration of 1.64 mg/mL. The molecular weights of the Lst-HDD dimer and monomer were estimated from the power law calibration curve using bovine serum albumin (Sigma-Aldrich, St. Louis, MO, USA), lysostaphin and lysozyme (AppliChem Panreac, Darmstadt/Barcelona, Germany/Spain) as standards.

### 3.3. Catalytic Activity

The catalytic activity of lysostaphin and Lst-HDD was determined by the reaction with pentaglycine. Pentaglycine peptide (sc-471644A, Santa Cruz Biotechnology Inc, Dallas, TX, USA) was dissolved in formic acid at a concentration of 400 mM, diluted to 8 mM in 20 mM HEPES buffer and neutralized with sodium hydroxide to pH 7.5. Equal amounts of pentaglycine solution and 2 µM lysostaphin or Lst-HDD in 20 mM HEPES buffer, pH 7.5 were mixed, leading to the final concentrations of 4 mM pentaglycine and 1 µM enzyme. The mixture was separated into 20 µL aliquots and incubated at 37 °C. The aliquots (*n* = 3) were taken at 2–3 h intervals and frozen at −20 °C to stop the reaction. To estimate the extent of pentaglycine cleavage, the aliquots were thawed at 50 °C, mixed with 100 µL of 0.4% ninhydrin in 80% DMSO/20% water, incubated at 85 °C for 15 min and mixed with 200 µL of water. After that, 100 µL of the resulting solutions were transferred to a 96-well plate and the absorbance was measured at iMark microplate reader (Bio-Rad Laboratories Inc, Hercules, CA, USA) at 595 nm. The increase in the optical density followed a linear trend during the first 8 h of reaction, and these data were used to calculate the reaction rate. The experiment was performed three times.

### 3.4. Minimum Inhibitory Concentration

The Minimum inhibitory concentration (MIC) was measured by standard microplate dilution procedure. Fresh overnight culture of *S. aureus* ATCC 29,213 was sampled from Brain Heart Infusion agar (Sifin Diagnostics, Berlin, Germany) plate and suspended in saline to an optical density corresponding to 0.5 McFarland. The bacterial suspension was diluted 300 times in Mueller-Hinton broth (Sifin Diagnostics, Berlin, Germany) with 2% NaCl and 0.1% BSA to an approximate density of 5 × 10^5^ CFU/mL. Then 90 µL of this bacterial suspension was transferred to a 96-well plate (Costar 3599, Corning, Corning, NY, USA), followed by 10 µL of serial two-fold dilutions of lysostaphin or Lst-HDD. The plate was incubated at 37 °C and 400 rpm for 20 h in a plate shaker (PST-60 HL plus, Biosan, Riga, Latvia), and MIC was determined as the lowest dilution of the protein that resulted in the absence of visible turbidity.

### 3.5. Staphylolytic Activity

The staphylolytic activity of the proteins was determined by monitoring the turbidity of staphylococcal cell suspension exposed to different concentrations of lysostaphin or Lst-HDD. Fresh overnight culture of *S. aureus* ATCC 29,213 was sampled from the BHI agar plate, inoculated into Mueller-Hinton broth with 2% NaCl and incubated overnight at 37 °C and 100 rpm. After that, the bacterial culture was vigorously vortexed for 1 min, spun down at 4000 g for 5 min, resuspended in TBS (50 mM Tris-HCl, 150 mM NaCl, pH 7.5), spun down again and resuspended in TBS with 0.1% BSA. The turbidity of the suspension was adjusted to 4.0 McFarland (~1.2 × 10^9^ CFU/mL) and 180 µL of the suspension was transferred to the wells of a 96-well plate. The plate was incubated at 37 °C and 400 rpm for 10 min, and 20 µL of various concentrations of lysostaphin or Lst-HDD were added to the wells. The turbidity of the suspension was monitored for 1 h at 37 °C with slow shaking, with measurements taken at 1 min intervals at the wavelength of 550 nm using Multiscan FC microplate reader (Thermo Fischer Scientific, Waltham, MA, USA). Measurements for each protein concentration within the experiment were performed in triplicate, and the experiment was performed three (lysostaphin) or two (Lst-HDD) times. To compare the staphylolytic activity between the proteins, the data were processed as follows. For every protein concentration, the time required to decrease the turbidity of the suspension by 50% (half-clearing time) was determined, and the relationship between the protein concentration and the half-clearing time was approximated by a power law function. This calibration curve was then used to estimate the half-clearing time for 50 nM of protein.

### 3.6. Anti-lysostaphin Antibody Production

Lysostaphin antiserum was obtained in rabbits. Briefly, two Chinchilla rabbits were subcutaneously immunized with 150 µg lysostaphin in complete Freund adjuvant at five sites, followed by booster immunization by the same amount of lysostaphin in incomplete Freund adjuvant after the two-week interval, and three subsequent intramuscular booster injections administered at one-week intervals starting three weeks after the first booster injection. The blood was collected by ear venipuncture one week after the last booster immunization, the serum was isolated and stored at −20 °C in 50% glycerol.

Murine ascitic fluid containing anti-lysostaphin monoclonal antibodies 2F9 was obtained from the State Research Center for Applied Microbiology and Biotechnology (Obolensk, Russia), deposition number H-29 [39].

The experiment was approved by N.F. Gamaleya Research Center biomedical ethics committee, protocol №15 16.10.2018.

### 3.7. Pharmacokinetics

The pharmacokinetic parameters of lysostaphin and Lst-HDD were estimated after single bolus intravenous injections in rats. Two groups of female Wistar rats (*n* = 4 per group) weighing ~300 g received bolus injection of 0.5 mg of lysostaphin or Lst-HDD in 0.5 mL saline in the tail vein. The blood was sampled from the tail vein at 25 min, 40 min, 1 h, 2 h, and 4 h time points in the group that received lysostaphin and 25 min, 40 min, 1 h, 2 h, 4 h, and 6 h time points in the group that received Lst-HDD. Plasma was separated from blood and stored at −80 °C until analysis. The residual concentration of lysostaphin and Lst-HDD in rat plasma was measured using sandwich ELISA. The 96-well plates (Costar 3590, Corning, Corning, NY, USA) were coated with rabbit lysostaphin antiserum diluted 1/500 in the carbonate-bicarbonate buffer, pH 9.7 overnight at +4 °C. After coating, the plate was washed twice with TBS with 0.05% Tween-20 (TBST) and blocked with 1% BSA in TBS for 3 h at 37 °C. After blocking, the plate was washed twice with TBST and plasma samples diluted 1/120 (lysostaphin) or 1/100 (Lst-HDD) in TBS, along with spiked calibration samples in the same matrix were added. The plate was incubated with plasma samples overnight at +4 °C. After that, the plate was washed twice with TBST, the ascitic fluid containing 2F9 monoclonal antibodies diluted 1/300 in TBST with 1% BSA was added and the plate was incubated overnight at +4 °C. The plate was then washed four times with TBST and HRP-conjugated secondary anti-mouse antibodies (IMTEK, Moscow, Russia) diluted in TBST with 1% BSA was added. The plate was incubated for 3 h at +37 °C, washed four times with TBST, the TMB substrate was added and the plate was incubated for 25 min at +37 °C. An equal volume of 2 M sulfuric acid was added to quench the reaction and the optical density was measured at 450 nm using an iMark microplate reader.

Terminal half-life and area under the curve (AUC) were calculated according to the non-compartmental approach as described in [40]. To calculate the terminal half-life of lysostaphin, data from the four last time points corresponding to the linear portion of the curve in log-linear coordinates were used. To calculate the terminal half-life of Lst-HDD, data from the three last time points were used. To determine the AUC, the concentrations were extrapolated to zero level using the calculated half-lives, and AUC was calculated by trapezoid rule starting from the first time-point.

To calculate the theoretical concentration of proteins in rat plasma after the injection, the average rat blood volume of 64 mL/kg [41] was used, and plasma was taken to amount ~50% of blood by volume.

The experiment was approved by N.F. Gamaleya Research Center biomedical ethics committee, protocol №15 16.10.2018.

### 3.8. Statistical Analysis

All results are presented as mean ± standard deviation. The statistical significance of the differences in the terminal half-lives and AUCs was determined by two-tailed Student’s *t*-test with unequal variances using Real Statistics Resource Pack add-on for Microsoft Excel (Release 4.7, copyright (2013–2017) Charles Zaiontz, www.real-statistics.com).

## 4. Conclusions

In the present study, we constructed a non-covalently dimerized version of antibacterial lysin lysostaphin and tested the influence of dimerization on its activity and pharmacokinetics. Unlike previously reported Cpl-1 dimer, lysostaphin dimerization decreased its bacteriolytic activity. We speculate that it might be caused by the ‘one-use’ property of lysostaphin and faster depletion of free active dimer due to its tighter binding to the cell wall debris of lysed bacterial cells. The plasma terminal half-life of dimerized lysostaphin increased two-fold, while AUC increased three-fold. This is in line with the results obtained with Cpl-1, although the magnitude of the effect is much smaller in our case. Thus, although dimerization turned out to be beneficial for the pharmacokinetic characteristics of lysostaphin, this strategy of increasing plasma half-life is not universal for antibacterial lysins, as it can decrease the bacteriolytic activity. The difference in the effect of the dimerization on the bacteriolytic activity of lysostaphin and Cpl-1 may be explained by natural dimerization propensity of Cpl-1, while lysostaphin normally exists in monomeric form. The main limitation of this study is the inability to demonstrate the mechanism responsible for the increase in the half-life and AUC of Lst-HDD. Presumably, the slower elimination results from the increased molecular weight due to dimerization. However, the simultaneous increase in the net positive charge and possible non-specific interactions of the dimerization domain with endogenous proteins may influence the clearance rate as well. The magnitude of such influences remains undefined. Nevertheless, to the best of our knowledge, this is the first study reporting the activity and pharmacokinetics of an antibacterial lysin dimerized through an additional domain.

## Abbreviations

AUC	Area under the curve
BCA	Bicinchoninic acid
BHI	Brain heart infusion
CFU	Colony forming units
DTT	Dithiothreitol
ELISA	Enzyme-linked immunosorbent assay
HRP	Horseradish peroxidase
IL-1	Interleukin 1
IPTG	Isopropyl thiogalactoside
MALDI	Matrix-assisted laser desorption/ionisation
MIC	Minimum inhibitory concentration
PEG	Polyethylene glycol
SEC	Size exclusion chromatography
TBS	Tris-buffered saline
TBST	Tris-buffered saline with 0.05% Tween 20
TMB	3,3′-5,5′-Tetramethylbenzidine
VEGF	Vascular endothelial growth factor

## References

[1] Smith R.A., M’Ikanatha N.M., Read A.F. (2015). Antibiotic Resistance: A Primer and Call to Action. Health Commun..

[2] Sabtu N., Enoch D.A., Brown N.M. (2015). Antibiotic resistance: What, why, where, when and how?. Br. Med. Bull..

[3] Santajit S., Indrawattana N. (2016). Mechanisms of Antimicrobial Resistance in ESKAPE Pathogens. BioMed Res. Int..

[4] Cetinkaya Y., Falk P., Mayhall C.G. (2000). Vancomycin-Resistant Enterococci. Clin. Microbiol. Rev..

[5] Gajdács M. (2019). The Continuing Threat of Methicillin-Resistant Staphylococcus aureus. Antibiotics.

[6] Sheu C.C., Lin S.Y., Chang Y.T., Lee C.Y., Chen Y.H., Hsueh P.R. (2018). Management of infections caused by extended-spectrum β–lactamase-producing Enterobacteriaceae: current evidence and future prospects. Expert Rev. Anti. Infect. Ther..

[7] El Chakhtoura N.G., Saade E., Iovleva A., Yasmin M., Wilson B., Perez F., Bonomo R.A. (2018). Therapies for multidrug resistant and extensively drug-resistant non-fermenting gram-negative bacteria causing nosocomial infections: a perilous journey toward ‘molecularly targeted’ therapy. Expert Rev. Anti-Infect. Ther..

[8] Gajdács M. (2019). The Concept of an Ideal Antibiotic: Implications for Drug Design. Molecules.

[9] Schillaci D., Spanò V., Parrino B., Carbone A., Montalbano A., Barraja P., Diana P., Cirrincione G., Cascioferro S. (2017). Pharmaceutical Approaches to Target Antibiotic Resistance Mechanisms. J. Med. Chem..

[10] Spengler G., Kincses A., Gajdács M., Amaral L. (2017). New Roads Leading to Old Destinations: Efflux Pumps as Targets to Reverse Multidrug Resistance in Bacteria. Molecules.

[11] Assis L.M., Nedeljković M., Dessen A. (2017). New strategies for targeting and treatment of multi-drug resistant Staphylococcus aureus. Drug Resist. Updat..

[12] Dickey S.W., Cheung G.Y.C., Otto M. (2017). Different drugs for bad bugs: antivirulence strategies in the age of antibiotic resistance. Nat. Rev. Drug Discov..

[13] Pastagia M., Schuch R., Fischetti V.A., Huang D.B. (2013). Lysins: the arrival of pathogen-directed anti-infectives. J. Med. Microbiol..

[14] Sharma U., Vipra A., Channabasappa S. (2018). Phage-derived lysins as potential agents for eradicating biofilms and persisters. Drug Discov. Today.

[15] São-José C. (2018). Engineering of Phage-Derived Lytic Enzymes: Improving Their Potential as Antimicrobials. Antibiotics.

[16] Jun S.Y., Jang I.J., Yoon S., Jang K., Yu K.-S., Cho J.Y., Seong M.-W., Jung G.M., Yoon S.J., Kang S.H. (2017). Pharmacokinetics and Tolerance of the Phage Endolysin-Based Candidate Drug SAL200 after a Single Intravenous Administration among Healthy Volunteers. Antimicrob. Agents Chemother..

[17] Cassino C., Murphy M.G., Boyle J., Rotolo J., Wittekind M. Results of the First in Human Study of Lysin CF-301 Evaluating the Safety, Tolerability and Pharmacokinetic Profile in Healthy Volunteers. Proceedings of the 26th European Congress of Clinical Microbiology and Infectious Diseases.

[18] Tang L., Meibohm B. (2006). Pharmacokinetics of Peptides and Proteins. Pharmacokinetics and Pharmacodynamics of Biotech Drugs: Principles and Case Studies in Drug Development.

[19] Walsh S., Shah A., Mond J. (2003). Improved Pharmacokinetics and Reduced Antibody Reactivity of Lysostaphin Conjugated to Polyethylene Glycol. Antimicrob. Agents Chemother..

[20] Loeffler J.M., Djurkovic S., Fischetti V.A. (2003). Phage Lytic Enzyme Cpl-1 as a Novel Antimicrobial for Pneumococcal Bacteremia. Infect. Immun..

[21] Rodríguez-Cerrato V., García P., Huelves L., García E., Del Prado G., Gracia M., Ponte C., López R., Soriano F. (2007). Pneumococcal LytA autolysin, a potent therapeutic agent in experimental peritonitis-sepsis caused by highly β-lactam-resistant Streptococcus pneumoniae. Antimicrob. Agents Chemother..

[22] Abdelkader K., Gerstmans H., Saafan A., Dishisha T., Briers Y. (2019). The Preclinical and Clinical Progress of Bacteriophages and Their Lytic Enzymes: The Parts are Easier than the Whole. Viruses.

[23] Entenza J.M., Loeffler J.M., Grandgirard D., Fischetti V.A., Moreillon P. (2005). Therapeutic Effects of Bacteriophage Cpl-1 Lysin against Streptococcus pneumoniae Endocarditis in Rats. Antimicrob. Agents Chemother..

[24] Kokai-Kun J.F., Chanturiya T., Mond J.J. (2007). Lysostaphin as a treatment for systemic Staphylococcus aureus infection in a mouse model. J. Antimicrob. Chemother..

[25] Swierczewska M., Lee K.C., Lee S. (2015). What is the future of PEGylated therapies?. Expert Opin. Emerg. Drugs.

[26] Zhang F., Liu M., Wan H. (2013). Discussion about Several Potential Drawbacks of PEGylated Therapeutic Proteins. Biol. Pharm. Bull..

[27] Resch G., Moreillon P., Fischetti V.A. (2011). PEGylating a bacteriophage endolysin inhibits its bactericidal activity. AMB Express.

[28] Cuesta A.M., Sainz-Pastor N., Bonet J., Oliva B., Alvarez-Vallina L. (2010). Multivalent antibodies: when design surpasses evolution. Trends Biotechnol..

[29] Sytkowski A., Lunn E. (1998). Human erythropoietin dimers with markedly enhanced in vivo activity. Proc. Nat. Acad. Sci. USA.

[30] Kontermann R.E. (2011). Strategies for extended serum half-life of protein therapeutics. Curr. Opin. Biotechnol..

[31] Resch G., Moreillon P., Fischetti V.A. (2011). A stable phage lysin (Cpl-1) dimer with increased antipneumococcal activity and decreased plasma clearance. Int. J. Antimicrob. Agents.

[32] Kokai-kun J.F. (2012). Lysostaphin: A silver bullet for staph. Antimicrobial Drug Discovery: Emerging Strategies.

[33] Gurnon D.G., Whitaker J.A., Oakley M.G. (2003). Design and Characterization of a Homodimeric Antiparallel Coiled Coil. J. Am. Chem. Soc..

[34] The European Committee on Antimicrobial Susceptibility Testing Breakpoint tables for interpretation of MICs and zone diameters. Version 9.0, 2019. http://www.eucast.org.

[35] Mitkowski P., Jagielska E., Nowak E., Bujnicki J.M., Stefaniak F., Niedziałek D., Bochtler M., Sabała I. (2019). Structural bases of peptidoglycan recognition by lysostaphin SH3b domain. Sci. Rep..

[36] Rennke H.G., Patel Y., Venkatachalam M.A. (1978). Glomerular filtration of proteins: Clearance of anionic, neutral, and cationic horseradish peroxidase in the rat. Kidney Int..

[37] Boksha I.S., Lavrova N.V., Grishin A.V., Demidenko A.V., Lyashchuk A.M., Galushkina Z.M., Ovchinnikov R.S., Umyarov A.M., Avetisian L.R., Chernukha M.I. (2016). Staphylococcus simulans recombinant lysostaphin: Production, purification, and determination of antistaphylococcal activity. Biochemistry.

[38] Shevchenko A., Tomas H., Havliš J., Olsen J.V., Mann M. (2007). In-gel digestion for mass spectrometric characterization of proteins and proteomes. Nat. Protoc..

[39] Vetchinin S.S., Pavlov V.M., Galkina E.V., Vakhrameeva G.M., Grishaeva N.S., Mokrievich A.N., Djatlov I.A. (2014). Strain of Hybrid Cultured Animal Cells Mus Musculus 2f9-Producer of Monoclonal Antibodies Specific for Lysostaphin and Inhibiting Its Lytic Activity. Patent.

[40] Gabrielsson J., Weiner D. (2012). Non-compartmental Analysis. Computational Toxicology: Volume I, Methods in Molecular Biology.

[41] Diehl K.H., Hull R., Morton D., Pfister R., Rabemampianina Y., Smith D., Vidal J.M., Van De Vorstenbosch C. (2001). A good practice guide to the administration of substances and removal of blood, including routes and volumes. J. Appl. Toxicol..

